# Optimal care at the end of life (OPAL): study protocol of a prospective interventional mixed-methods study with pretest-posttest-design in a primary health care setting considering the view of general practitioners, relatives of deceased patients and health care stakeholders

**DOI:** 10.1186/s12913-019-4321-9

**Published:** 2019-07-15

**Authors:** Kambiz Afshar, Gabriele Müller-Mundt, Katharina van Baal, Sophie Schrader, Birgitt Wiese, Jutta Bleidorn, Stephanie Stiel, Nils Schneider

**Affiliations:** 1 Institute for General Practice, Hannover Medical School, Carl-Neuberg-Straße 1, 30625 Hannover, Germany; 2 Institute of General Practice and Family Medicine, Jena University Hospital, Bachstraße 18, 07743 Jena, Germany

**Keywords:** Primary palliative care, General practice, Health care services, Complex intervention, SPICT

## Abstract

**Background:**

At the end of life, about 85–90% of patients can be treated within primary palliative care (PC) provided by general practitioners (GPs). In Germany, there is no structured approach for the provision of PC by GPs including a systematic as well as timely identification of patients who might benefit from PC, yet. The project “Optimal care at the end of life” (OPAL) focusses on an improvement of primary PC for patients with both oncological and non-oncological chronic progressive diseases in their last phase of life provided by GPs and health care services.

**Methods:**

OPAL will take place in Hameln-Pyrmont, a rural region in Lower Saxony, Germany. Target groups are (a) GPs, (b) relatives of deceased patients and (c) health care providers. The study follows a three-phase approach in a mixed-methods and pre-post design. In phase I (baseline, t_0_) we explore the usual practice of providing PC for patients with chronic progressive diseases by GPs and the collaboration with other health care providers. In phase II (intervention) the Supportive and Palliative Care Indicators Tool (SPICT) for the timely identification of patients who might benefit from PC will be implemented and tested in general practices. Furthermore, a public campaign will be started to inform stakeholders, to connect health care providers and to train change agents. In phase III (follow-up, t_1_) we investigate the potential effect of the intervention to evaluate differences in the provision of PC by GPs and to convey factors for the implementation of SPICT in general practices.

**Discussion:**

The project OPAL is the first study to implement the SPICT-DE regionwide in general practices in Germany. The project OPAL may contribute to an overall optimisation of primary PC for patients in Germany by reducing GPs’ uncertainty in initiating PC, by consolidating their skills and competencies in identifying patients who might benefit from PC, and by improving the cooperation between GPs and different health care stakeholders.

**Trial registration:**

The study was retrospectively registered at the German Clinical Trials Register (Deutsches Register Klinischer Studien; trial registration number: DRKS00015108; date of registration: 22th of January 2019).

## Background

At the end of life, only 10–15% of patients need specialized palliative care (PC) whereas the majority of people can be treated within primary PC provided by general practitioners (GPs) [1, 2].

Primary PC is considered as the basis of all PC concepts although there is no universally accepted definition [3]. Main characteristics of primary PC are: care is provided by health care providers who are not specialists in PC, patient‘s situation is less complex than in a specialized PC setting, and benefits are not linked to specific structural requirements [3].

Specific fields of action in primary PC are defined in the German guideline [3] as follows:

- Treatment of symptoms and support in problems of little to moderate complexity in all four dimensions (physical, psychological, social and spiritual),
- Communication, clarification of therapeutic objectives and coordination of care,
- Involvement of specialized PC when indicated.

Although PC in Germany underwent a remarkable development in the past 30 years, there are still considerable challenges and barriers. A major barrier in the provision of PC is the systematic and timely identification of patients who might benefit from PC, especially in old patients with multimorbidity and/or chronic progressive diseases as e.g. congestive heart failure or chronic obstructive pulmonary disease [4, 5]. Illness trajectories and needs of these patients are characterized by uncertainty, ambiguity and a high variability [6–8].

Therefore, it is often unclear to define at what time PC is indicated and should be considered in these patients [9–11].

Different studies emphasize the necessity for a timely and systematic identification strategy for patients with chronic progressive diseases who might benefit from PC [12–16].

Internationally, different tools to support the identification of patients in need for PC have been developed and implemented successfully in different care settings [16–19]. One of these tools is the Supportive and Palliative Care Indicators Tool (SPICT™) [20]. The SPICT™ is a clinical tool first developed in 2010 as a collaborative project between National Health Service Lothian and the University of Edinburgh Primary Palliative Care Research Group. It is part of the Gold Standards Framework in the United Kingdom and supports the identification of people with deteriorating health and with potential unmet PC needs [21]. Until today, it is translated in eight different languages and used in over 28 different countries worldwide [22]. The German version of the SPICT™ (SPICT-DE) was systematically translated and adjusted in 2017 [23]. SPICT-DE is a single-page clinical tool comprising three parts: part one with general indicators (e.g. unplanned hospital admission, poor performance status), part two with clinical indicators of life-limiting conditions (e.g. cancer, kidney, liver, or neurological diseases) and part three with recommendations on concrete PC actions and care planning. Pilot studies in different settings revealed that SPICT-DE is considered to be practical and helpful to identify patients who might benefit from PC [23, 24]. Yet, it is unclear if the systematic application of SPICT-DE in general practices results in an optimisation of care for patients with chronic progressive diseases in the last phase of their lives. In this study, we refer to the broader definition of end of life care defined as the last two years of life according to the European Association of Palliative Care [25].

### Aims

Overall goal of the study “Optimal care at the end of life” (OPAL) is to improve GPs’ care of patients with chronic progressive diseases in the last phase of their lives taking into account the perspectives of relatives and different health care providers.

Further objectives are (a) to increase awareness for PC needs and actions in primary PC provided by GPs and (b) to support the systematic identification of patients who might benefit from PC. These goals shall be achieved by an implementation of SPICT-DE in general practices the region Hameln-Pyrmont in Lower Saxony, Germany. The study concept focusses on two different levels: (1) primary outpatient PC provided by GPs and (2) collaboration of different health care and PC providers (e.g. nursing services, nursing homes, hospitals, and specialists).

### Research questions

The main research question of this study is:

- Does the application of the SPICT-DE improve the care of patients with chronic progressive diseases in the last phase of their lives?

Further research questions are as follows:

- Is SPICT-DE accepted by GPs as a clinical tool and applicable in their practice routine to provide care for patients with chronically progressive diseases?
- Does the application of SPICT-DE increase GPs’ awareness and sensitivity for PC situations?
- Does the application of SPICT-DE result in PC actions in general practice?

## Methods and design

OPAL is a three-year project (01.07.2018–30.06.2021) funded by the innovation fund of the Federal Joint Committee (Grant No.: 01VSF17028).

### Setting

OPAL is based on a regional approach and will be conducted in the primary care setting of the administrative district Hameln-Pyrmont in Lower Saxony. Hameln-Pyrmont (area 796 km^2^, 148.296 inhabitants) is located in a region between Hannover, Hildesheim and East Westphalia-Lippe with a mixed structure of eight urban and rural communities. Furthermore, Hameln-Pyrmont is one of currently 35 so-called health regions in Lower Saxony [26] aiming to promote the cooperation of regional structures in health care. Hameln-Pyrmont shows regional differences concerning the availability of health care services and facilities. OPAL takes advantage of already established networks as for instance coordination centers, working groups and steering committees. The project will be embedded in existing infrastructure and benefit from well-established platforms (e.g. health conferences; “Gesundheitskonferenz” or “Gesundheit im Dialog”). These interdisciplinary events intend to support multidisciplinary and intersectoral care involving different health care stakeholders.

### Study design

OPAL is a prospective interventional mixed-methods study with pretest-posttest-design. According to the model of stages by the Medical Research Council (MRC) for complex interventions, OPAL is planned as a MRC-Phase-I study [27, 28].

The development of the study design is based on international studies evaluating interventional concepts and on national programs for the improvement and integration of PC in primary care at the end of life [17, 29].

### Study phases

**Fig. 1:**
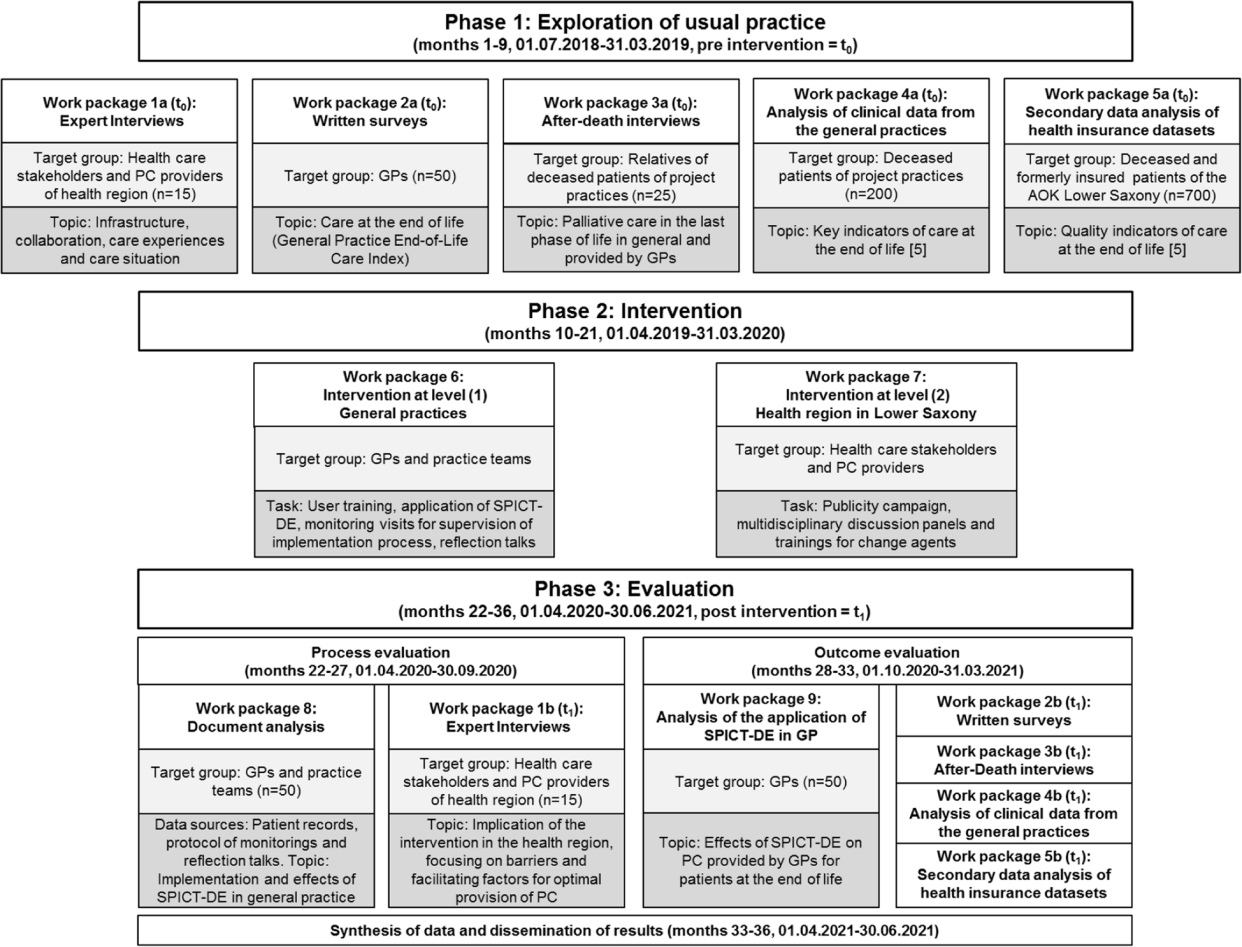
Design, phases, work packages and timeline of the study OPAL modelled after Campbell et al. [28, 30]

OPAL comprises three phases and focusses on two different levels: (1) primary PC provided by GPs and (2) PC provided by different health care providers in the health region Hameln-Pyrmont in Lower Saxony. Figure 1 outlines all phases, work packages and the corresponding timeline. Table 1 is a Gantt chart with information on study phases, work packages and detailed timeline.

**Table 1:**
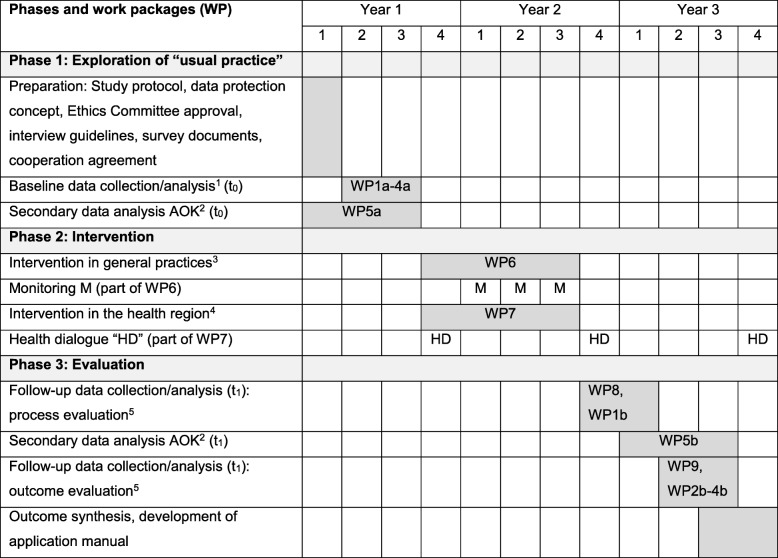
Gantt chart with information on study phases, work packages and timeline

^1^Baseline (t_0_): WP1a interviews with health care stakeholders (n = 15), WP2a written surveys with GPs (*n* = 50), WP3a after-death interviews with relatives of deceased patients (*n* = 25), WP4a analysis of clinical data of deceased patients from the 35 general practices (*n* = 200 or approx. 4 per project practice)

^2^Anonymised datasets of the AOK (Local Health Care Fund) from in defined period of time before (WP5a, baseline t_0_, Year 1, *n* = 700) and after (WP5b, follow-up survey t_1_, Year 3, n = 700) the intervention will be analysed

^3^Intervention in general practices (WP6): user training and application of SPICT-DE, monitoring visits for supervision of the implementation process and reflection talks

^4^Intervention at the level of the health region (WP7): Publicity campaign, multidisciplinary discussion panels and trainings for change agents (health dialogue, HD)

^5^Follow-up (t_1_): WP1b interviews with health care stakeholders (*n* = 15), WP2b written surveys with GPs (*n* = 50), WP3b after-death interviews with relatives of deceased patients (*n* = 25), WP4b analysis of clinical data of deceased patients from of the 35 general practices (*n* = 200 or approx. 4 per project practice)

### Phase I: exploration of usual practice (9 months)

In phase I, we explore the usual practice of care for patients with chronic progressive diseases in the last phase of life (baseline data collection, t_0_). Phase I comprises the following five work packages:

- Work package 1a: Guided expert interviews (*n* = 15) with health care stakeholders and PC providers of different settings focusing on infrastructure, collaboration, care experiences, and care situation.
- Work package 2a: Standardized written survey with GPs (*n* = 50) of 35 different single or group practices to give an insight into organization and quality of PC for patients in the last phase of life. For this purpose, we will use the German version of the General Practice End-of-Life Care Index (GP-EoLC-I) [31].
- Work package 3a: Guided after-death interviews with relatives (*n* = 25) of deceased patients of the 35 general practices focusing on how they experienced and perceived PC in the last phase of life in general and provided by GPs.
- Work package 4a: Analysis of clinical data of patients with chronic progressive diseases of the 35 collaborating general practices who died within a defined period of time. The analysis is based on key indicators (e.g. chemotherapy for cancer patients in the last month of life, number of hospitalisation and treatment days in the last six months of life, number of patients who died in the hospital) to evaluate the quality of care at the end of life [5].
- Work package 5a: Secondary data analysis of patients who died in a defined period of time before the intervention and were insured members of the Local Health Care Fund (AOK) in Lower Saxony. The analysis is based on key indicators to evaluate the quality of care at the end of life [5].

### Phase II: intervention (12 months)

The intervention in phase II will take place at two levels: (1) in the 35 general practices and (2) in the health region Hameln-Pyrmont, including different health care stakeholders and PC providers. Phase II comprises the following two work packages:

- Work package 6 (intervention at the level of general practices): The study team will visit each GP and their practice teams for a standardized training in using SPICT-DE. To ensure a common understanding of the term “palliative care”, a definition based on the German guideline [3] and on the World Health Organization [32] will be given to each GP in hard copy. The user training will be performed (duration: approximately 15 min) to illustrate the application of the SPICT-DE according to the recommendations of the “Guide to use SPICT-DE in the community” [22] and using two exemplary case vignettes. GPs then will be asked to apply the SPICT-DE in daily practice during a period of twelve months in any patient that would visit the practice or would be seen in domiciliary visit regardless of their place of living (e.g. at home, nursing home or care facility) and that would meet the following inclusion criteria: age ≥ 18 years with at least one oncological or non-oncological chronic progressive disease according to the SPICT-DE. Patients with a specialized out- or inpatient PC approach or residents of hospices will be excluded. For each patient meeting the inclusion criteria, GPs will be asked to highlight all applicable indicators of the SPICT-DE. In order to monitor the indicators chosen by GPs, check boxes will be added for each indicator and recommended PC action listed in the SPICT-DE. There will be the opportunity to mention any additional action as a free-text answer as well. The decisions and procedures regarding the patients for whom SPICT-DE has been used will be documented in the practice documentation system. Monitoring visits will be performed quarterly for supervision of the implementation process and for reflection on the application of the SPICT-DE in daily practice routine. Both the monitoring visits and the reflection talks will be minuted for process evaluation (see phase III).
- Work package 7 (intervention at the level of the health region): The implementation of the SPICT-DE in general practices will be complemented by a publicity campaign (including flyers, newsletters and a local press campaign), multidisciplinary discussion panels and trainings for change agents in the health region. These activities shall inform stakeholders and health care providers at the interfaces of PC about OPAL and its intervention in general practices. The intervention at this level ought to optimise the collaboration of different health care providers and PC professionals as well as the intersectoral provision of care for patients at the end of life.

### Phase III: evaluation (15 months)

In phase III, process and outcome evaluation will give an insight into barriers, facilitating factors, effects of the intervention, and reveal information on possibilities for sustainable maintenance.

- Work package 8: This work package is part of the process evaluation. Protocols from the monitoring visits and reflection talks in the 35 general practices during the intervention (phase II) will be analysed. Results will give further information about the implementation process of the SPICT-DE in general practice and its practicability as well as efficacy in daily GPs’ practice.
- Work package 9: This work package is part of the outcome evaluation. GPs will be asked to fill out a single-page semi-structured questionnaire with six questions concerning alterations in the patients’ situation and the occurrence of any critical incidences for every patient after the first application of the SPICT-DE. A critical incidence is defined as e.g. acute crises in the disease progression, unplanned hospital admissions, changes in therapy, care, and living environment as well as death of patients. Furthermore, GPs will be asked to indicate for each patient if and if so which PC actions as recommended by the SPICT-DE were initiated. Analysis of the indicators of the SPICT-DE and of initiated PC actions will give further information on the effects of the intervention and the handling of SPICT-DE in daily practice.
- Work packages 1b-5b (follow-up, t_1_): These work packages will be performed in an equivalent way as described for the work packages 1a-5a of phase I (baseline data collection, t_0_): guided expert interviews (*n* = 15), standardized written survey with GPs (*n* = 50) of the 35 general practices, guided after-death interviews with relatives (*n* = 25) of deceased patients of the 35 general practices, analysis of clinical data of patients with chronic progressive diseases of the 35 collaborating general practices who died within a defined period of time, and secondary data analysis of patients who died in a defined period of time after the intervention and were insured members of the Local Health Care Fund (AOK) in Lower Saxony. Results of these work packages will allow the pre-post comparison.

### Study population and recruitment

OPAL is addressed to four different target groups: (1) health care stakeholders, (2) GPs and their practice teams, (3) relatives of deceased patients with chronic progressive diseases, and (4) deceased patients with chronic progressive diseases.

1. Health care stakeholders (*n* = 15) at the interfaces of PC and GP care such as e.g. nursing services, hospitals, hospices, and medical specialist PC teams will be informed about the project and invited by letter/mail and by telephone to take part in OPAL.
2. GPs (*n* = 50) of 35 different general practices being registered in Hameln-Pyrmont will be informed about the project and invited by letter/mail and by telephone to take part in OPAL. GPs and their practice teams will receive an allowance for their participation in the study.
3. Relatives (*n* = 25) of deceased patients of the 35 general practices are eligible when they meet the following inclusion criteria: age ≥ 18 years and a direct contact to the deceased patient in the last phase of their life (independent of sex and gender). Recruitment follows the principle of purposive sampling. The relatives are recruited by the GPs and their practice teams. To assure data protection, relatives will contact the study team when interested or their contact data will only be given to the study team by GPs and their practice teams after prior informed consent and permission. This procedure has been applied successfully in other studies [33–35].
4. Patients (age ≥ 18 years, independent of sex and gender) with oncological and/or non-oncological chronic progressive diseases who died during a defined period of time before (t_0_) and after (t_1_) the intervention while being treated by one of the collaborating GPs will be included for further data analysis using standardized questionnaires. Furthermore, members (age ≥ 18 years, independent of collaborating GP practices as well as sex and gender) of the Local Health Care Fund AOK in Lower Saxony who died in a defined period of time before (t_0_) and after (t_1_) the intervention will be included in the secondary data analysis based on key indicators to evaluate the quality of care at the end of life [5]. An exclusion criterion for both groups of deceased patients is sudden death, which cannot be related to a chronic progressive disease (e.g. traffic accident, heart attack without prior diagnosis of coronary heart disease).

### Sample size calculation

1. Main target of the intervention are GPs in Hameln-Pyrmont (work package 2a and 2b). A total of 105 GPs is registered in 75 general practices in this region. Based on the recruitment rates of previous studies a participation of 50 GPs out of 35 general practices can be assumed. Results of a power analysis reveal a medium effect of the intervention with 50 GPs participating in OPAL. A sample size of 50 GPs has approx. 80% power to prove an average pre-post difference of four points in the GP-EoLC-I with assumption of 10 points standard deviation (paired t-test, two-sided 5% significance level) [31].
2. The second target group is formed by relatives of deceased patients. Each independent *n* = 25 relatives are interviewed before and after the intervention phase (work package 3a and 3b).
3. For work package 1a and 1b, *n* = 15 experts/health care stakeholders at the interfaces of PC and GP care (see above) are supposed to be interviewed twice, in both pre and post intervention phase, as a third target group.
4. The fourth target group consists of deceased patients from the participating general practices (work package 4a and 4b). The collection of clinical data is scheduled for six months before and six months after the intervention phase. Gágyor et al. determined an average of 15 cases of death in each general practice per year (standard deviation 7.3, range 4.36) [36]. Consequently, a total of 500 cases of death in twelve months and respectively 250 cases of death in six months in 35 general practices are expected. Thereof about 80% had chronic progressive diseases, so that data from *n* = 200 patients would be available for the analyses in each observation period.
  With a total of 2.8 million members, the AOK in Lower Saxony represents one-third of all statutory health insured people in Lower Saxony [37]. The health region Hameln-Pyrmont counts about 35.000 members. There are 35.000 cases of death of insured members in one year while the targeted health region sums about 700 cases of death within the insured members of the AOK in Lower Saxony [37].

### Outcomes

Table 2 gives an overview of the objectives, outcome parameters, data collection, data sources as well as the work packages and the timeline in OPAL.

**Table 2:** Objectives, outcome parameters, data collection/sources, work packages and timeline

Objective	Outcome parameter	Data collection/source	Work package
Optimised care at the end of life	Primary outcome: General Practice End of Life Care Index (GP-EoLC-I): clinical practice and organization of palliative care	Standardized written surveys with GPs	WP2a t_0_ WP2b t_1_
	Secondary outcome: Situation of care, shared decision-making, advance care planning, consideration of therapeutic preferences, preferred place to live and to die.	Guided interviews with relatives of deceased patients (after-death interviews)	WP3a t_0_WP3b t_1_
	Secondary outcome: Indicators for (palliative) care at the end of life based on the Bertelsmann foundation’s fact check 2015: ▪ Chemotherapy for cancer patients in the last month of life, ▪ Insertion of percutaneous endoscopic gastrostomy tube in the last three months of life, ▪ Number of hospitalisation and treatment days in the last six months of life, ▪ Number of patients with general outpatient PC treatments according to the Uniform Value Scale, ▪ Number of prescriptions for specialized outpatient PC and number of first prescriptions within the last three days of life, ▪ Average duration of general and specialized outpatient PC treatments, ▪ Number of patients who died in the hospital.	Clinical data of deceased patients of general practices	WP4a t_0_WP4b t_1_
		Comparative analysis of AOK datasets: Lower Saxony in total vs. Hameln-Pyrmont	WP5a t_0_ WP5b t_1_
Application of the SPICT-DE	Number of patients in whom the SPICT-DE was applied to identify a PC situation	Patient records	WP9
GPs’ awareness and sensibility for PC situations	Number of patients considered eligible for PC; number of patients with general outpatient PC treatments; number of prescriptions for specialized outpatient PC	Patient records AOK datasets	WP8 WP5a t_0_ WP5b t_1_
Effectiveness and practical consequences for GPs	Number of PC actions and interventions for identified patients (e.g. review of current treatment and medication, considering referral for specialist PC, advance care planning.)	Patient records	WP8 WP9

t_0_: pre intervention (baseline), t_1_: post intervention

Primary outcome is an optimised care at the end of life delivered by GPs using the German version of the GP-EoLC-I (work package 2a/b). It contains 13 items about clinical aspects (four-stage Likert scale: 4 = in every case, 3 = in most cases, 2 = in some cases, 1 = rarely or never) and 12 items about organizational parameter of end of life care. The GP-EoLC-I showed good internal consistency (clinical care Cronbach’s α: 0.847; organization Cronbach’s α: 0.684). The overall index is built by summation of all 25 items and a sum score between 25 and 100 (Cronbach’s α: 0.850) [31].

Secondary outcome is an optimised care at the end of life seen from the perspective of bereaved relatives (work package 3a/b) and displayed in data of deceased patients (work package 4a/b). Amongst others, decisive aspects will concern the overall care situation, the involvement in decision making, in defining care goals and advance care planning, the preferred place of living at the end of life, and preferred place of death.

Optimisation of care for patients at the end of life will also be assessed considering approved key indicators to evaluate the quality of end of life care (work package 5a/b) [5]:

- Chemotherapy for cancer patients in the last month of life,
- Insertion of percutaneous endoscopic gastrostomy tube in the last three months of life,
- Number of hospitalisation and treatment days in the last six months of life,
- Number of patients with general outpatient PC treatments according to the German Uniform Value Scale (Einheitlicher Bewertungsmaßstab, EBM),
- Number of prescriptions for specialized outpatient PC and number of first prescriptions within the last three days of life,
- Average duration of general and specialized outpatient PC treatments,
- Number of patients who died in the hospital.

Further outcome parameters according to the research questions are as follows:

- Application of the SPICT-DE in general practice analysing the number and data of patients in whom the SPICT-DE was applied to identify a PC situation (work package 9),
- GPs’ awareness for PC situations analysing the number of patients who will be considered eligible for PC, the number of patients with general outpatient PC treatments and the number of prescriptions for specialized outpatient PC (work packages 5a/b and 8),
- Effectiveness and practical consequences for GPs are evaluated through the number of initiated PC interventions for patients with chronic progressive diseases as recommended by the SPICT-DE, e.g. review of current treatment and medication, considering referral for specialist PC, advance care planning (work packages 8 and 9).

### Ethics and data protection

The study was approved by the Ethics Committee of the Hannover Medical School in August 2018 (No.: 8038_BO_K_2018). Written informed consent will be obtained from all GPs, relatives of deceased patients and health care experts by the study team prior to any study procedure.

Each participant will be assigned with an individual code in order to pseudonymise the related data. The code list will be archived and locked separately from the data collection documents. Only study members will have full access to study material. Furthermore, GPs will list each deceased patient included for testing and assign them with an individual ID. That list remains in the general practices and will be inaccessible for the study team. Patient data collected and stated by the GPs in the questionnaires will be given anonymously to the research team so that patients’ identity will be fully preserved. Essential amendments to the study protocol or the interventions will be reported to the Ethics Committee of the Hannover Medical School for approval.

### Data analysis

Qualitative data: Interviews will be audio recorded, pseudonymised, transcribed, and analysed using the software MAXQDA. All transcripts will be analysed using qualitative content analysis as described by Mayring [38] and by Meuser and Nagel especially for the analysis of expert interviews [39].

Quantitative data: For data entry and data analyses the Statistical Package for the Social Sciences (SPSS) will be used. For the evaluation of the difference in the GP-EoLC-I between pre and post intervention phase paired t-test will be applied. To compare parameters of patients in the pre and in the post intervention phase different tests for independent samples will be performed depending of the underlying distribution of the outcome parameters (e.g. Mann-Whitney-U-test, χ^2^-test, and unpaired t-test). Secondary data analysis of claims data of the Local Health Care Fund (AOK) in Lower Saxony will be performed using descriptive analyses.

## Discussion

### Provision of PC using SPICT-DE

International research indicates that SPICT™ can increase the awareness of professional health care providers for PC situations and support the systematic initiation of PC actions in both patients with chronic oncological and non-oncological progressive diseases who might benefit from PC [14–16].

The systematic development, refinement and testing of SPICT-DE formed a process of overall five years and was successfully completed by using a multiprofessional and participatory approach [23]. In particular, involving members of the target group of potential users in the development and testing process was important to increase acceptance of SPICT-DE before its implementation. Preliminary research showed that SPICT-DE is a helpful and practical tool to support the identification of patients who might benefit from PC [24].

### Innovative potential and benefit

OPAL is the first study to implement the SPICT-DE regionwide in general practices in Germany. The application, acceptance and efficacy of SPICT-DE as a practical, patient-oriented identification and decision-making tool for patients with chronic progressive diseases who might benefit from PC will be evaluated in mutual experience exchange between GPs and their practice teams, relatives and health care providers and services.

OPAL focusses its intervention at two different levels: (1) general practices and (2) health care providers and services in Hameln-Pyrmont, Lower Saxony. Thus, interprofessional and intersectoral cooperation, communication and coordination of PC for patients at the end of life will be enhanced and optimised.

The introduction of SPICT-DE in the general practice setting in Germany might change the usual identification strategy and possibly increase GP’s awareness in providing PC for patients with different chronic progressive diseases. The tool may contribute to consolidating skills and competencies of GPs in identifying patients with potential PC needs and increase their confidence in initiating PC. The results may also contribute to the overall improvement of care for patients and family caregivers as well as to the optimisation of primary PC by GPs and other health care providers in Germany. Furthermore, the dissemination of the SPICT-DE across the regions may lead to its integration in national strategies to promote PC in the community.

### Expected results

OPAL may contribute to an overall optimisation of care for patients with chronic progressive diseases in the last phase of their lives. GPs play a key role in the systematic identification of patients who might benefit from PC in the primary care setting [3]. The implementation of SPICT-DE allows for a systematic and potentially timely identification of these patients and for initiating specific PC actions which may positively affect the further care of patients and their relatives, e.g. enhancing patient autonomy, participatory decision-making, preventing futile treatment at the end of life [20].

Furthermore, considering the desires, needs and requirements of relatives may identify and avoid overburdening (e.g. due to domestic care or psychosocial burden) preventatively. Thereby, OPAL contributes to a patient-centered and family-oriented care.

At the same time, equitable access to PC may be optimised: so far, especially patients with oncological diseases benefit from PC concepts [40]. The provision of PC is also dependent on the social and educational level. In this study we focus on the needs and desires of patients and their relatives regardless of diagnosis or social background.

The intervention in OPAL may also contribute to an efficient use of resources. European health economic studies indicated that a systematic and structured provision of PC might achieve cost savings for the overall healthcare system [41].

A potential cost saving effect may be due to avoiding therapeutic treatment and unnecessary hospital admission [1, 42, 43]. We expect that the implementation of SPICT-DE may reduce or at least not increase supply costs in the last phase of life whilst improving the quality of care.

### Challenges

Empirically, a selection bias must be taken into account for research projects in general practices. It is much likely, that especially the scientifically interested GPs of the well-established teaching and research practices will be willing to participate. In OPAL, all GPs of the health region will be invited for participation using existing infrastructure of both districts and benefit from well-established information events, coordination centers, working groups and steering committees. The project will be embedded in these existing infrastructures.

For the after-death-interviews relatives of deceased patients of the general practices will be asked for participation. Due to data privacy protection the recruitment and initial invitation will be performed by the GPs and their practice teams and not by the study team. Relatives’ expense and burden will be decreased to a minimum. Relatives will set the time and place of the interviews which will be performed without time pressure and will be stopped at any time upon their request. Experience of prior studies showed that a trustful relationship between the study team and GPs as well as between GPs and the relatives is crucial to promote the recruitment strategy [44, 45]. Nonetheless, a selection bias cannot be fully excluded.

### Dissemination and implementation

OPAL has a high potential for sustainable maintenance of the implementation of the SPICT-DE in the primary care setting. If SPICT-DE is applied successfully and evaluated positively, the implementation strategy can be extended to other care regions and federal states of Germany. It can be assumed that the application of SPICT-DE may lead to a systematic initiation of PC actions and a more accurate prescription of specialized PC in the primary care setting. To promote further implementation of SPICT-DE, different formats will be suitable: e.g. PC in undergraduate medical education, postgraduate further education in PC, specialized training in PC, online courses and quality circles. Integrating the results of OPAL in education and training for physicians, nursing staff and other health care professionals will increase the awareness for PC for patients with chronic progressive diseases.

Publication in peer-reviewed open access journals and presentation at national as well as international PC conferences will disseminate results and make them accessible for a broader audience. There are no publication restrictions. For communication of the trial results to the participants, a newsletter will be send to GPs as the main target group after each study phase.

Following a very practical approach, the transferability of interventions in OPAL can be considered as promising. The results of OPAL can easily be transferred to other care regions and federal states in Germany. Different patient groups, relatives, GPs and health care stakeholders involved in the provision of PC at the end of life may benefit from the innovation content of OPAL.

## Data Availability

Data collected during study application are available from the corresponding authors on reasonable request. Datasets from the Local Health Care Fund Lower Saxony were generated and analysed during the theoretical phase of this study and are not publicly available due to regulations of data privacy protection.

## Abbreviations

AOK: Local Health Care Fund
EBM: Uniform Value Scale (Einheitlicher Bewertungsmaßstab)
GP: General practitioners
GP-EoLC-I: General Practice End of Life Care Index
MRC: Medical Research Council
OPAL: Optimal care at the end of life
PC: Palliative care
SPICT-DE: German version of the Supportive and Palliative Care Indicators Tool
SPICT™: Original Version of the Supportive and Palliative Care Indicators Tool
